# Physical-Mechanical Properties of Stone Masonry of Gjirokastër, Albania

**DOI:** 10.3390/ma14051127

**Published:** 2021-02-27

**Authors:** Enea Mustafaraj, Erion Luga, Marco Corradi, Antonio Borri, Ylber Muceku, Aleksandra Zharkalli

**Affiliations:** 1 Department of Civil Engineering, EPOKA University, 1039 Tirana, Albania; emustafaraj@epoka.edu.al (E.M.); eluga@epoka.edu.al (E.L.); azharkalli14@epoka.edu.al (A.Z.); 2 Department of Mechanical & Construction Engineering, Northumbria University, Newcastle upon Tyne NE1 8ST, UK; 3 Department of Engineering, University of Perugia, 06125 Perugia, Italy; antonio.borri@unipg.it; 4 Institute of Geosciences, Energy, Water and Environment, 1024 Tirana, Albania; y.muceku@geo.edu.al

**Keywords:** masonry, historic construction materials, mechanical testing, earthquake engineering

## Abstract

In addition to reinforced concrete and steel buildings, a large part of the existing building stock in Europe is made of stone masonry. Prediction of the structural behavior requires the development of a systematic material characterization of the mechanical properties and structural details (units, arrangement, bonding, inter-connection). This study aims to analyze the mechanical and physical behavior of building stones in the historical city of Gjirokastër, Albania, known also as the Stone City. A thorough investigation of the regional stone quarries was performed, and the collected samples were cut into regular prismatic specimens for further analysis. The experimental campaign consisted of the determination of flexural strength and compressive strength, water absorption, porosity, specific gravity as well as structural analysis of the masonry material, using the MQI (Masonry Quality Index) method. The test results showed that there is a large scattering in the values of the mechanical and physical stone properties such as compressive strength varying from 20 to 115 MPa and flexural strength from 8 to 25 MPa. However, the analysis of the masonry material revealed a satisfactory structural performance, based on a frequent, systematic respect of the good construction practices (i.e., the rules of the art) in Gjirokastër.

## 1. Introduction

**Figure 1:**
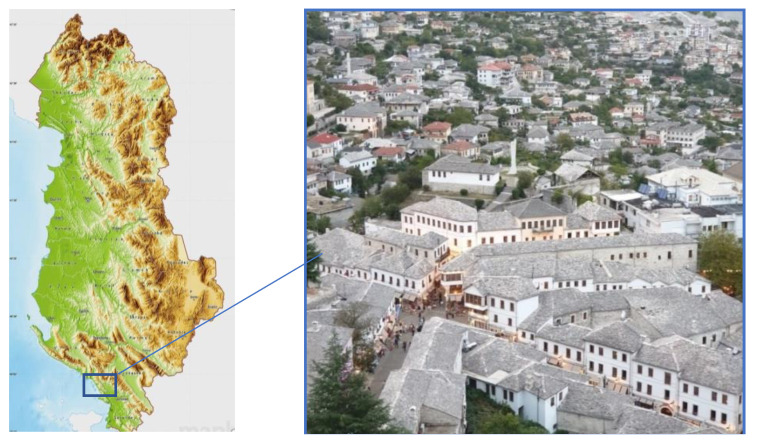
Location of the city (**left**) and aerial view of the old city center (**right**).

The city of Gjirokastër is located in southern Albania, situated in a valley between the *Mali i Gjerë* mountain and *Drino* river, at 300 meters above sea level (Figure 1). Known also as the Stone City, in 2005, Gjirokastër was announced a UNESCO (The United Nations Educational, Scientific and Cultural Organization) protected site defined as “a unique example of a well-preserved” Ottoman town [1] with a building stock mostly dating from the 17th and 18th centuries.

**Figure 2:**
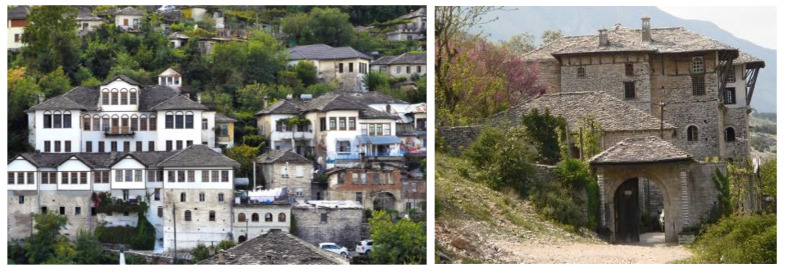
Typical historic masonry constructions of Gjirokastër.

Nowadays, in Gjirokastër there are 590 monuments, which can be grouped into two main categories according to their importance. In the 1st category, there are about 56 monuments while at the 2nd category 540. Overall, near the city around 1200 stone buildings [2] are found. The typical buildings consist of a high stone block structure up to five-story high (Figure 2). Single family dwellings are generally smaller compared to multi-family residential buildings which are of a considerable size. For their construction, different building materials such as stone, wood, clay, glass, gypsum, plaster, binding mortar, goat hair, etc., are used. In general, most of these materials have a local origin. From the natural materials that are present in these buildings the most commonly used are stone and wood.

Stone has not only been used for the construction of some of the most important monuments and structures but, at the same time, all the old streets are made of stone because of its strength, hardness, abrasion resistance, and durability. In general, in the whole range of constructions, there have been involved native highly skilled stonemasons, with the intent of preserving the characteristic architecture and building technology of the old town. Moreover, being so abundant in this region, stone has always been an affordable and cost-effective building material.

An important past research [2] about the buildings of Gjirokastër, their classification according to general type and building characteristics, was carried out several years after the city was listed by the Albanian Heritage and Conservation Authorities. This is a study in which an effort is made to provide the characteristics of the “fortified Gjirokastër housing” considered as a type of Albanian heritage building stock, analysis of the origin and its evolution [3].

At the same year, Kamberi [4] investigated the building techniques of Gjirokastër buildings of the 18-19th centuries. The main construction materials were also studied by [5,6], mostly by visual assessment and observations. Other studies were focused on the identification of the architectural features and restoration of important buildings [7,8,9,10] and ethnographic values of the city [11,12], byzantine churches and monasteries [13,14], and other important values. Nevertheless, there is a lack of research about the mechanical characteristics of the construction materials used in Gjirokastër. 

Over the past decades, several studies on Albanian heritage structures [15] and assessment and retrofitting of unreinforced masonry buildings have been conducted by the authors [16,17,18].

In Europe, on the other hand, several examples on characterization of physical and mechanical characteristics of construction materials can be mentioned: dry stone heritage materials and limestone from Portugal [19,20], freshwater limestones used in Bosnia and Herzegovina [21] and limestones from Italy [22]. Techniques involve on-site testing [23] and adoption of non-destruction techniques [24,25].

Moreover, in Italy, following 1998 and 2009 earthquakes, in Greece, in the Balkan countries and in several other territories where the seismic hazard is significant, a large amount of research has been devoted in academia to the use of composite materials as a method to increase the in-plane shear capacity of stone masonry wall panels [26]. These are typically epoxy-bonded to the masonry walls. Recently, in consideration of the unsatisfactory long-term behavior of the bonding and low compatibility with masonry material, epoxy resins have been replaced with inorganic matrices, i.e., mortars. In this latter method, composite materials (typically fiberglass meshes or steel cords) are embedded into a mortar coating to be applied on each wall faces [25,27,28].

However, the design of reinforcement interventions depends on the structural behavior of the unreinforced, original masonry, including not only the mechanical characteristics of the constituent materials (stone and mortar), but also the masonry texture and arrangement (stone shape and dimensions, type of wall, wall thickness, etc.,). As a consequence, many studies have been published to assess the structural response of unreinforced stone masonry [29,30,31,32], highlighting the frequent unsatisfactory response against the seismic action.

Stone masonry buildings are particularly vulnerable to earthquakes, and it is well accepted that a preliminary assessment of the mechanical performance of stone masonry buildings is an important prerequisite for any future action. This information is critical not only for design, but also for the policymaker and governmental agencies dealing with the risk-analysis of the masonry building stock. Destructive in situ structural testing would be the best method to measure the mechanical properties of the stone masonry. This is typically carried out on wall panels cut off from the load-bearing walls of the buildings. Diagonal tension [33], compression [34], and shear-compression [35] tests can provide very useful data about the structural response of masonry (strengths, moduli, strains, yielding behavior, etc.,). However, these tests are highly invasive, expensive, and destructive: in many situations, conservation bodies do not permit such types of tests on the listed buildings.

Nevertheless, structural engineers need to know these properties for their calculations, as mentioned above. To meet this need, we opted to estimate the masonry mechanical characteristics using the technique proposed by Borri et al. [36,37,38], known as the Masonry Quality Index (MQI). This is a visual method of assessment of the masonry mechanical properties based on the masonry mechanical given in the Italian Building Code, (NTC)-2018 [39,40] and calibrated on a large database (over 120 test results) of destructive tests carried out by the authors in the last 25 years.

## 2. Aim

This experimental work aims to analyze the main mechanical and physical properties of the traditional building stones in the historic city of Gjirokastër. For this reason, a thorough investigation of the regional quarries was performed, and the collected samples were cut into regular prismatic specimens for further analysis. The experimental campaign consisted of the determination of flexural strength and compressive strength, water absorption, porosity, the specific gravity, as well geological and petrographic characteristics.

Moreover, classification of the principal types of residences and masonry typologies is presented. Based on the stone load bearing wall characteristics, the mechanical properties and the expected structural response under seismic actions are investigated using the Masonry Quality Index (MQI).

**Figure 3:**
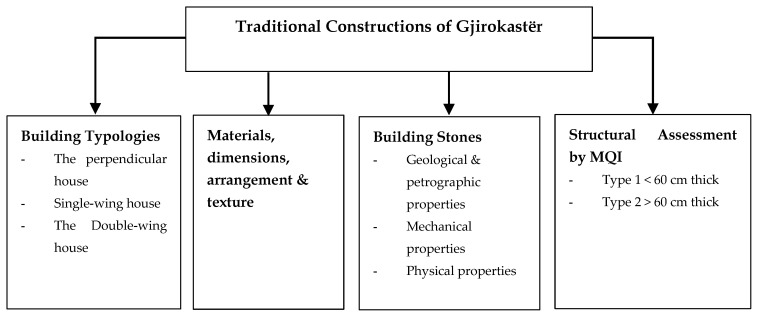
Experimental campaign flowchart.

As a result, these data can be used for a detailed assessment of individual buildings depending upon their masonry typologies. The assessment procedure is presented in the Figure 3.

## 3. Gjirokastër case study 

### 3.1. Materials, Dimensions, Arrangement, and Texture

In Gjirokastër, stone was used from the foundations of the buildings till the roofs and can be identified in six primary groups (according to the names used locally by stone crafters): 1. *White limestone*, 2. *Semi- crystalized white limestone*, 3. *Black stone – silicate*, 4. *Grey stone*, 5. *River stone*, and 6. *Porous stone of Peshkëpi*.

The white limestone was mainly used for structural walls in the buildings: this was taken either from the construction site, or from areas near the city in quarries around Gjirokastër, in Lazarat, Derviçan, Goranxi, and Grapsh. The white limestone can be found on-site in three different colors: (a) white matte (the color is absorbed from magnesium oxide), (b) red (the color is released by the oxide of iron), (c) Gushëpëllumbi (pigeon neck) (the color is released by the oxide of copper).

White matte is a stone with many possible applications; this type of stone varies from 20 to 200 mm in thickness and it is considered as the most popular. This was mainly used for construction of the cobbled road due to its high strength and good abrasion resistance. The stone is dense with very low porosity and has a very good resistance against freezing and thawing. Moreover, since it is a material that can be easily cleft to particular shapes, it is used for decorations.

The red stone is mainly used for the characteristic roofs of the city. It is extracted in 15–30 mm thick layers. In addition, because of its pleasant color, this stone has a large use for decoration in cobbled roads, arched doorway, or in inner design.

The *Gushëpëllumb* stone is a mixture of yellow, white, red, and violet colors. This stone is widely used in architectural applications for walls, doors, as well as for decoration. 

The other group of the building stones of Gjirokastër is the dark stones. They are made of hydroxides, quarts manganese, and phosphates. In this group, there are two types of stone: the black and the grey stones. Both of them are used for building, floors, and pavements. The black stone is often used in cobbled streets of the city where together with the white matte and red stone it creates the unique street pavements of the historical city of Gjirokastër.

The black stone has a natural layering, which is formed in large blocks of varying thickness from 0.8 m to 1.2 m. It is either extracted from the area around the building, or from the surrounding area of the city, for example in Poliçan and Lunxhëri. The grey stone is used mostly by the people of Odrie.

The river stone is extracted in relatively large dimensions from the Drinos river and near the village of Kardhiq. When compared to other stones, this type is used less. It is commonly used for shared walls between *two buildings*. Sometimes in stonemasons it is combined with the black stone or concrete and is used for pavement purpose. On the other hand, the Peshkëpi stone is extracted in irregular shapes and it is used for decoration purposes as it has a very beautiful natural shape.

### 3.2. Classification of the Principal Types of Residences

The *City Tower* is one of the most developed dwellings typologies of the 18–19th centuries, only used in Gjirokastër. The early versions of these buildings were also applied in the cities of Berat, Krujë, and Shkodër. 

The most evident characteristic of this typology is the adaptation of the rational volume composition, development of mezzanine floor, unequal height distribution between stories, thick perimetral stone masonry walls (that served as a fortification), small openings, materials and positioning were closely connected with the folded terrain of the city. The main characteristic of the masonry buildings of Gjirokastër is the protective character: these are typically fortified constructions [2]. The principal constitutive parts of the typical buildings are the perimetral walls, "the outside *Oda* " (a separate structure from the building generally placed in the yard), *Katoli* (used for storing food), *Stera* (used as water tank, made of stones and plastered with impermeable mortar), the big *Oda* (or the guest room) etc. 

The main characteristic of the masonry buildings of Gjirokastër is the protective character: these are typically fortified constructions [2]. The development of the “City Tower” building typology is closely associated with the socio-economic changes occurring in the city of Gjirokastër. Based on the plan-volume composition criteria, the typical unreinforced stone masonry houses are categorized into three main groups [3]: 1. *Perpendicular Building*, 2. *One-wing Building*, 3. *Two-wings Building*.

#### 3.2.1. The Perpendicular Building

The base template for the other groups, is found in two-to-three story buildings often with a mezzanine floor. It is well-known for the enclosed volume and the defensive character, first seen during the 14th century and evolved to three-stories at the beginning of the 18th century.

**Figure 4:**
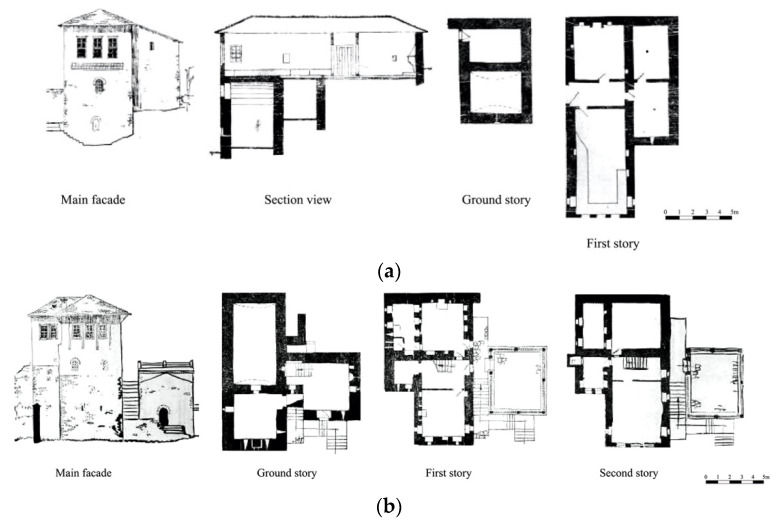
Examples of the perpendicular building: (**a**) two stories, (**b**) three stories (adapted from [3]).

The base is a rectangle with the longer sides perpendicular to the terrain. The connection between the stories was done by stone-made stairs (Figure 4). The volume of this dwelling is discontinuous with the upper floor, partly as ground floor lies of the back. On the ground floor there is the water deposit. The wider story accommodates the main building, and the guestrooms are located on top of it. The sanitary units are located by the side of the building. There is a clear differentiation of the functions of the stories. Over the years, the evolution of this building typology consisted of the addition of a third story. The new three-story building would be the base unit of further elaboration of this typology with distinctive functions: ground story serving for auxiliary services, the first floor for every-day living of family members, and the second story usually for guests. In the later evolved versions, this story would be used by the family members during the summertime as the spaces are bigger and the story height from 0.6 to 1. 6 m higher.

#### 3.2.2. The Single-Wing Building

**Figure 5:**
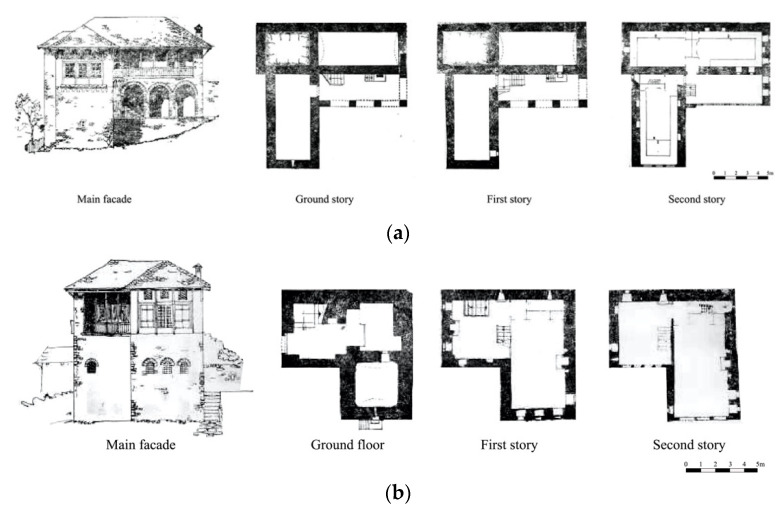

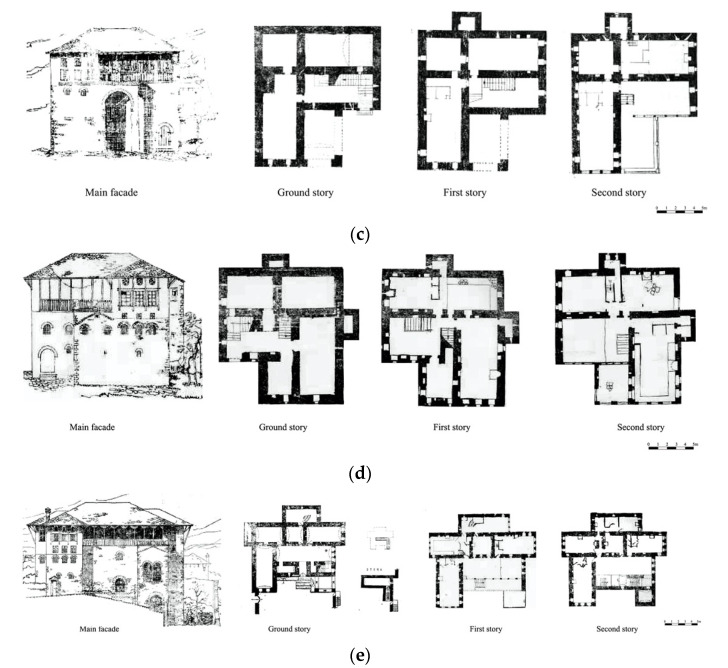
Examples of the single-wing building located at: (**a**) P. Xhixho St, n.15; (**b**) A. Toro St, N.20; (**c**) K.Bako St, n.17; (**d**) M. Bakiri St, n.45; (**e**) A. Frasheri St, n.6 (adapted from [3]).

The Single-wing building is the most common type of residence in Gjirokastër due to its versatility in terms of spaces, and volume adaptation. This typology is found mostly in the form of a three-story building. There are three sub-groups: the first one is characterized by the presence of the stairs inside of the buildings; the second group is known for the simple plan; and the third group, more irregular, is influenced by the morphology of the terrain as the volumes of the buildings are not well distributed (Figure 5).

#### 3.2.3. The Double-Wing Building

**Figure 6:**
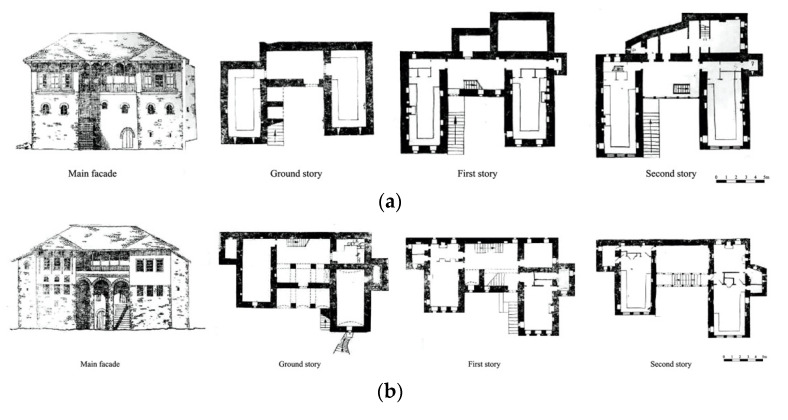

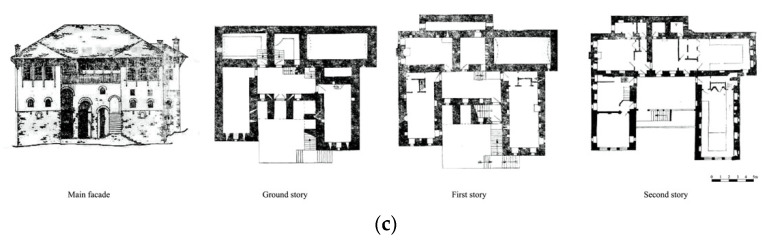
Examples of the double-wing building: (**a**) Babameto Building (1885), (**b**) Zekate Building (1812), (**c**) Skëndulaj Building (1823) (adapted from [3]).

This typology is one of the most featured versions of the buildings of Gjirokastër. The wings are two-story, and the central main part is three-story in height. It is a timely evolution of the other typologies (Figure 6).

### 3.3. Material, Dimensions, Stone Arrangement, and Texture

The foundations of the buildings in Gjirokastër are dug up to 130 cm deep into the ground. They are mostly made of dry stacked stone with no mortar, with big-sized stones or large pieces of rocks quarried around the city. This type of system allows for good drainage. On the other hand, the load bearing walls are made of white stone with a varying thickness of 60–75 cm. Whenever this kind of stone was not available, the black stone was used. The black stone is mainly found at *Pllakë* and *Pazari i Vjetër* neighborhoods.

The construction of the stone masonry walls is often made of three wythes (triple-leaf): the outer, the middle, and the outer wythe. The binding element for the stones of inner and outer wythe is either lime mortar or clay mortar. Regardless of the thickness of the wall, in the inner wythe, no mortar was typically used. One of the main advantages of this dry wall technique is the preventing of capillary actions inside the wall, thus, avoiding the risk of humidity and water infiltrations. According to the construction methods, the stone masonry can be categorized into two main groups:

1. *Thick Masonry*: with no anti-seismic timber ties, these buildings, generally single-story, are the oldest in the city, and are made of large stone blocks. During the 18–19th centuries, partially dry-stone walls were used for construction. The key characteristic is the inner-to-outer leaf transversal connection (known as header stone). This technique required a connection every four stones. When the cross-sectional thickness of the walls exceeds 60 cm, the wall is made of three wall leaves (triple-leaf wall). The outer and inner leaves were made of large stone blocks and the core leaf was typically filled with small stones. These walls were often reinforced with oak wood elements, internally installed during their assemblage in both longitudinal and orthogonal directions, creating a mesh that limits deformation and cracking. These walls were often reinforced with oak ties, on both faces of the walls, starting at 120 cm from the ground level, spaced every 80–110 cm centers connected by nails, creating a mesh that limits deformation and cracking.

2. *Thin Masonry:* This is used mainly for the partition walls in the upper stories. These walls are built of small stones bonded with mortar and have a thickness of no more than 12 cm.

### 3.4. Geological and Petrographic Characteristics

**Figure 7:**
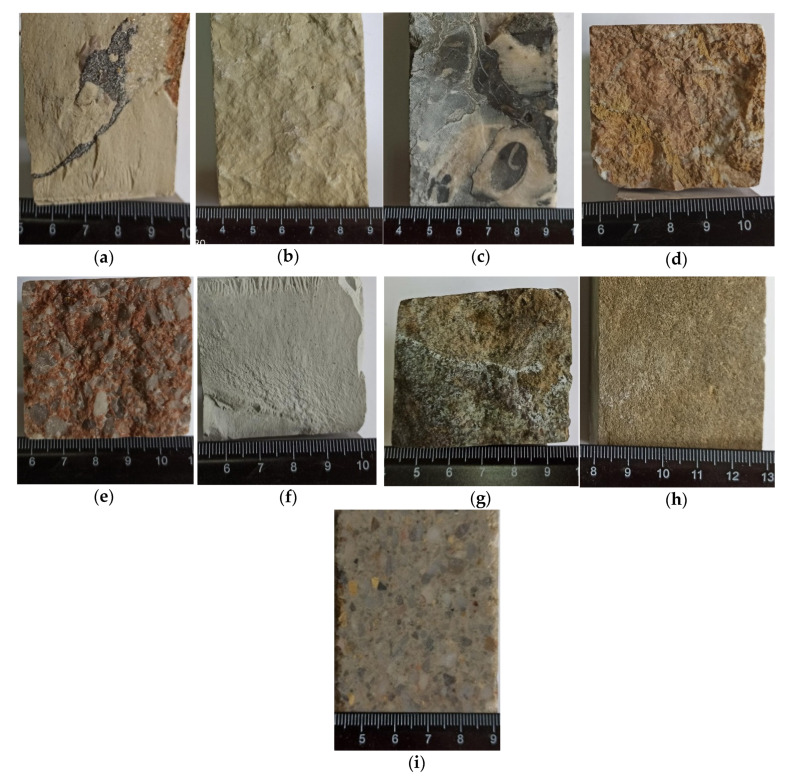
Thin bedded limestone: (**a**) micritic, (**b**) medium-grained, (**c**) limestones with rudist, (**d**) macro-grained; (**e**) conglobreccias and breccias; (**f**) dolomites (**g**) marl; (**h**) sandstones, (**i**) breccies [units: cm].

The stone quarries used for the construction of the buildings of Gjirokastër are found in the northern, western, and southern parts of the region. From the geological formation characteristics, the building stones are classified in the following types: i. The thin bedded limestones; ii. thick bedded to massive breccia’s limestones and conglobreccias limestones; iii. the bedded dolomites; iv. the thin bedded marls; v. the thin bedded sandstones. The pattern of each type of the limestones is shown in Figure 7.

#### 3.4.1. The Thin Bedded Limestones

Buildings made with this stone are located in northern, western, and southern part of Gjirokastër. They consist of thin strata 3–5 cm up to 20–50 cm. Many thin sections of limestones samples taken from different buildings were done during last years, were observed using a polarized microscope. From these studies, the limestones are divided in: 1. *Micritic limestones*: compact and hard rocks, dense and mainly light gray and milky color; 2. *Medium-grained limestones*: compact and hard rocks, dense and mainly wite color; 3. *Organogenic limestones with rudist*: characterized by hard rocks, very compact and black color; 4. *Macro-grained limestones*: medium to hard rocks, compact and dark grey and black color.

Considering the time of formation, the thin bedded limestones belong to Paleocene (Pg1) and Eocene (Pg2) series. They are generally characterized by micro fissures, randomly oriented and cemented with calcite. According to discontinuities the rock mass is generally affected by three system joints (cleavage), which are two vertical joints sets and a stratification joints with horizontal sets. The joints of vertical sets have a moderate spacing (1–2 mm to 5 mm). The limestones generally are composed of the calcite (90–99%) and dolomite impurity (1–10%). The other constituent’s minerals as clay, ancerites, aragonites, magnesites, and rodocrosites etc., are found in small quantities.

#### 3.4.2. Thick Bedded to Massive Conglobreccias and Breccias Limestones

They are mainly thick bedded to massive with varied and multicolored gravels and rubbles grains, having the mosaic view after the sawing. They are commonly hard rocks and have reddish color. The age of these formations is Lower Cretaceous (Cr1).

#### 3.4.3. The Bedded Dolomites

This type of building stones extend along the northern part of Gjirokastër. They are commonly thin to thick bedded limestones, grey and beige color. These rocks are softer than limestones, therefore they are easily seen. These deposits are found in Ftera village, Mali Gjërë. They consist of thin strata, from 5–10 cm to 20–40 cm. The age of these formations is Lower to Upper Cretaceous (Cr1-Cr2).

#### 3.4.4. The Bedded Marl’s Limestones

These formation are represented by medium to thick layers with glass textures, compact and reddish in color. The marl’s limestones are medium to hard rocks. The age of these formations is Paleocene (Pg1) and Eocene (Pg2) series.

#### 3.4.5. The Thin Bedded Sandstones

They are found in flysch’s formations of Lower Oligocene-Pg13, between claystones and siltstones layers. These deposit are stratigraphic break over Eocene and upper Cretaceous limestones. The sandstones are thin layers (3–5 cm to 10 cm thick), micro-granular, compact, and grey color. They are included in weak to medium group. 

## 4. Material Characterization and In Situ Test Results

### 4.1. Building Stones

**Figure 8:**
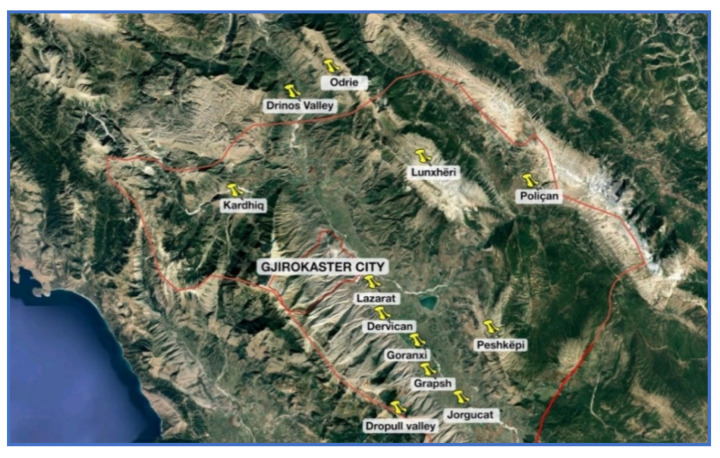
Locations of Gjirokastër in Albania and the quarries of stones.

From a field investigation, seven traditional quarry areas were identified within the region of Gjirokastër and some representative specimens were collected for laboratory testing. The collected specimens were shortly identified according to the following letter designation: D—Red Limestone collected from Dropulli valley, J—White limestone collected from Jorgucat village, G—Gushëpëllumbi (pigeon neck) limestone found also in Dropulli valley, L—Black silicate stone found in Lunxhëri and Poliçan, O—Grey sandstone collected in Odrie village, P—Porous stone of Peshkëpi, and K—Riverstone of Kardhiq and Drino river (Figure 8).

**Figure 9:**
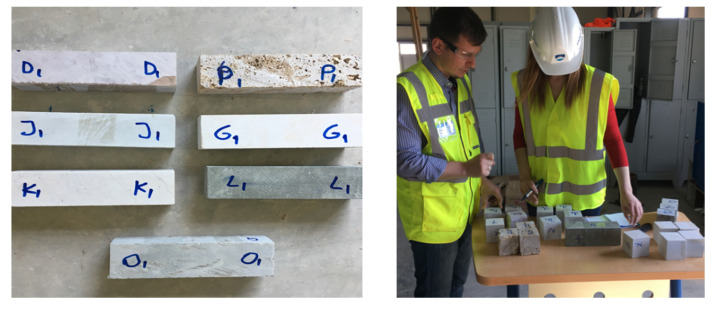
Representative stone prisms before testing.

The raw samples were cut into regular prismatic or cubic shape in accordance with the respective EN standard such as EN 12372 [41] for the flexural strength and EN 1926 [42] for the compressive strength. The produced specimens were used also for the determination of the physical properties. Table 1 and Figure 9 show the coding and preparation of some representative samples for testing.

### 4.2. Results

Compressive and flexural strength tests are the most common tests performed for the determination of the mechanical behavior of the building stones.

#### 4.2.1. Flexural Strength

The flexural strength test was performed in accordance with EN 12372 [41] using equation (1). According to this the thickness of the stone sample *h* should be between 25 mm and 100 mm, the total length *l* should be equal to six times the thickness, the width *b* should be between 50 mm and three times the thickness (50 mm ≤ *b* ≤ 3 *h*), and the distance between the supporting rollers *l* should be equal to five times the thickness.

(1) $$\sigma_{u}=\frac{3F_{f}}{2bh^{2}}l\;,$$

**Figure 10:**
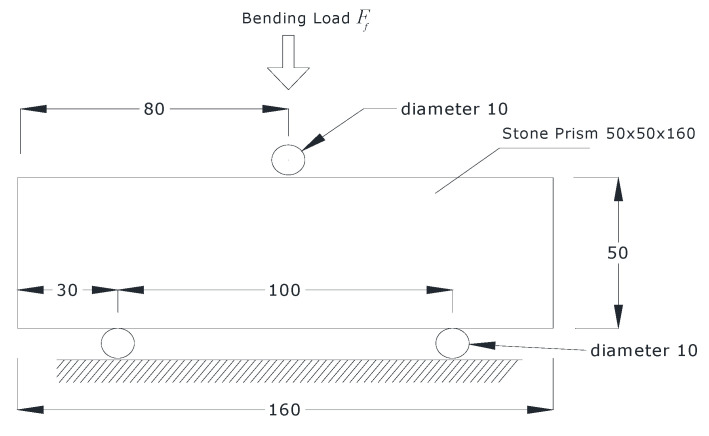
The schematic representation of the testing procedure (dimensions in mm).

Figure 10 shows the schematic view of the testing procedure. In this study, the cross section of the specimen was 50 × 50 mm. Three samples were tested for each stone type and the average flexural strength value was calculated.

#### 4.2.2. Compressive Strength

The compressive strength test was performed in accordance with EN 1926 [42]. It is defined that the test specimens were cut in cubes with (70 ± 5) mm or (50 ± 5) mm edges or right circular cylinders with diameter and height equal to (70 ± 5) mm or (50 ± 5) mm. In Figure 9 are presented the stone prisms before testing. For this study, the specimen cubes were (70 ± 5) mm in size. Three samples were tested for each stone type and the average compressive strength value was calculated. The compressive strength is calculated by the formula in equation 2, where F is the applied load in N and A is the resisting area in mm^2^.

(2) $$\sigma_{c}=\frac{F_{c}}{bh},$$

**Figure 11:**
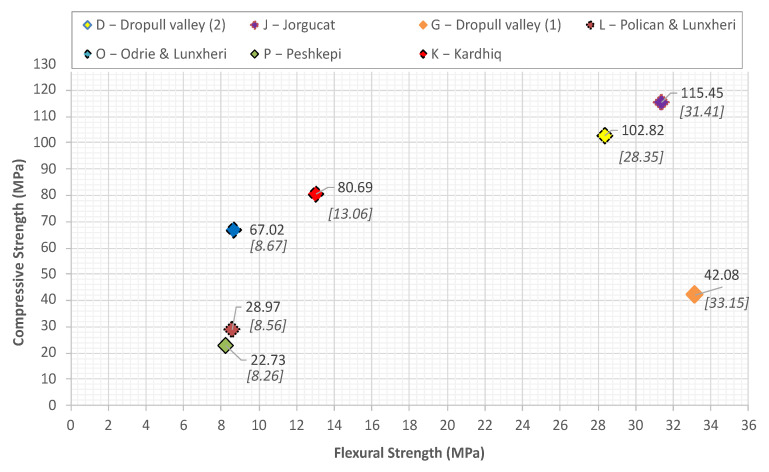
Compressive and flexural strengths of the tested specimens.

In Figure 11 are plotted the compressive strength and flexural strength of the tested specimens. According to the test results the flexural strength values vary from 8 to 33 MPa. The compressive strength test results showed that there is a large range in the values varying from 25 to 115 MPa. Physical properties test like open porosity and apparent density were performed according to EN 14617-1 [43], whereas the water absorption of the specimens was measured in compliance with EN 13755 standard [44].

#### 4.2.3. Physical Properties

Physical properties test like open porosity and apparent density were performed according to EN 14617-1 [34], whereas the water absorption of the specimens was measured in compliance with EN 13755 [35]. The following equations show the calculation of the physical properties.

*Open Porosity*:

(3) $$p\left(\%\right)=\frac{M_{ssd}-M_{dry}}{M_{ssd}-M_{w}}100,$$

*Apparent Density*:

(4) $$\rho *=\frac{M_{dry}}{M_{ssd}-M_{dry}},$$

*Water absorption*:

(5) $$W_{a}\left(\%\right)=\frac{M_{ssd}-M_{dry}}{M_{dry}}100,$$

where *M_w_* is the mass of the sample fully immersed in water, *M_ssd_* is the saturated surface dry mass of the sample, *M_dry_* is the completely dry mass of the sample.

**Figure 12:**
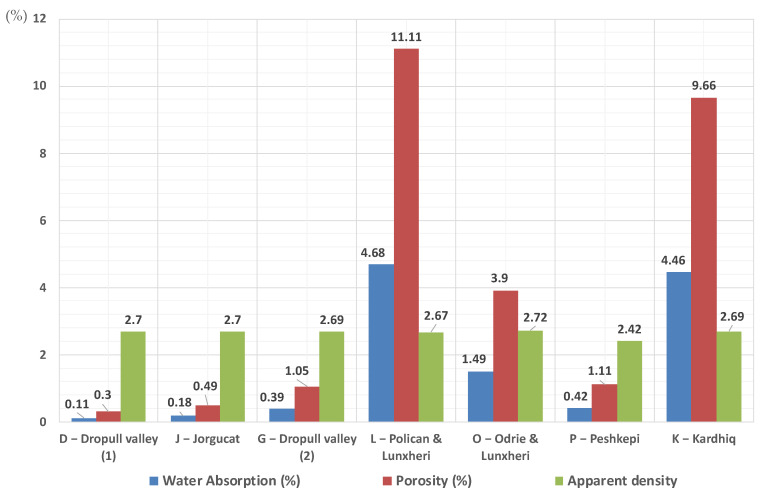
Physical properties of the tested specimens.

In Figure 12 are plotted the physical properties of the tested specimens. The porosity values ranged from 0.30 to 11.11%. On the other hand, it was observed that, as expected, the water absorption values were closely related to porosity, ranging from 0.11 to 4.68%.

## 5. Structural Assessment of the Masonry

**Figure 13:**
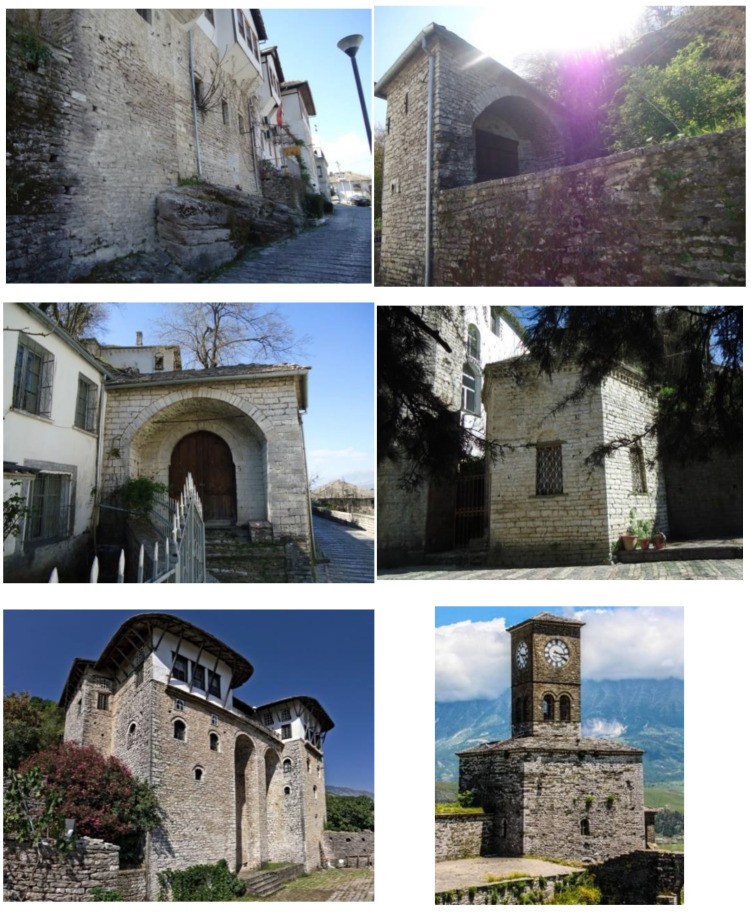
Several typical stone masonry buildings of Gjirokastër.

In this section, the mechanical properties and the expected structural response of the historic masonry constructions of Gjirokastër under the action of an earthquake have been evaluated. These properties are typically employed in commercially available modeling software (Ansys, Algor, SAP, etc.,) for numerical analyses and represent the fundamental parameters governing the structural behavior of a masonry building. In Figure 13 are presented some typical stone masonry buildings of Gjirokastër. 

Structural assessment of masonry is done using the MQI method which consists of a visual survey of the faces and the cross section of a wall. It considers seven critical parameters (mechanical characteristics and quality of the masonry units (acronym SM), dimensions of the masonry units (SD), shape of the masonry units (SS), level of connection between adjacent wall leaves (WC), horizontality of mortar bed joints (HJ), staggering of vertical mortar joints (VJ), quality of the mortar/interaction between masonry units (MM)).

**Figure 14:**
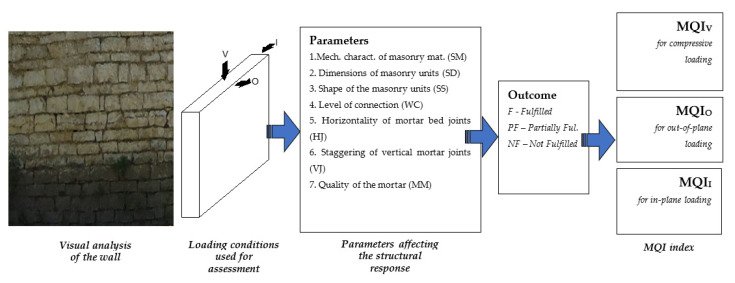
Summary of the MQI (Masonry Quality Index) method.

For each parameter, the visual analysis may result in three different outcomes: Fulfilled—F, Partially Fulfilled—PF, Not Fulfilled—NF. A chart of the method is summarized in Figure 14.

**Figure 15:**
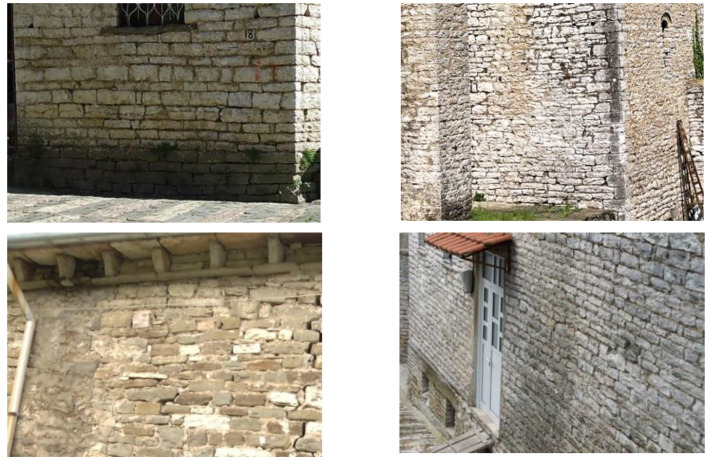
Typical stone walls of Gjirokastër.

The analysis of each parameter leads to a numerical value (for a total of 7 numerical results) based on its fulfillment category. The combination of the seven numerical values gives the value of MQI [40]. This is based on a wall panel and it is aimed at verifying if a wall complies with the “rules of the art” for the Gjirokastër historic walls (Figure 15). Based on this analysis, it is possible to calculate three numerical values. Because the structural response of a wall also depends on the loading condition, the values assigned to the seven parameters depend on the loading condition acting on the wall under consideration. Three loading conditions are considered in the MQI method: V (vertical loads), O (out-of-plane horizontal loads), and I (in-plane horizontal loads). Consequently, three different MQI values (MQI_V_, MQI_O_, and MQI_I_), can be calculated. The approach is to attribute different weights to the above parameters (between 0 and 3) based on the evidence that they affect the quality of the masonry with different degrees depending on the loading condition (Table 2). In case of fulfilment of all parameters of quality, the MQI index is 10 irrespective of the loading condition.

The different masonry typologies can be compared with the mechanical characteristics suggested by the Italian Building Code (NTC 2018) [39]. Numerous tests, carried out on-site by the authors to validate the method, have demonstrated that the index is able to provide useful information about the mechanical characteristics, and structural response in general, of the analyzed wall panel.

Most parts of the masonry buildings in Gjirokastër have been constructed using white limestone (letter designation J, Table 1) and soft stone (letter designation L). In the following paragraphs the MQI has been calculated for these two types of stone materials.

### 5.1. Wall Panels Type 1 (Cross Sectional Thickness <60 cm)

**Figure 16:**
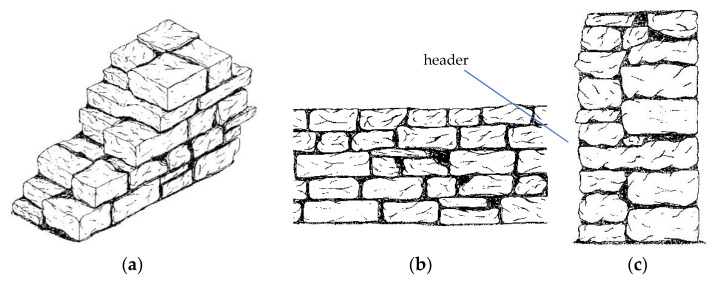
Sketches of a wall panel Type 1 (cross sectional thickness < 60 cm): (**a**) axonometric view, (**b**) front view, (**c**) wall cross section.

In this section, we consider masonry stone walls having a cross-sectional thickness up to 60 cm (Figure 16). Only load-bearing walls are used in the analysis. The MQI assessment of the walls gave the results shown in Table 3. This is based on the assumption of the use of a lime mortar for all studied walls. It is worth noting that masonry walls of Gjirokastër are properly assembled with regard to three critical “rules of the art”: 1. *the horizontality of the bed joints* (HJ parameter), 2. *the staggering of the head joints* (VJ parameter) and 3. *the worked shaped (parallelepiped) of the stone units* (SS parameter). It is also important to highlight that header stones (stone blocks placed transversally to the plane of the walls) were used in Gjirokastër in historic constructions. Their frequency in the walls is not high, but sufficiently enough to provide a connection between the wall leaves.

This is an important general observation, not common in historic masonry constructions in Southern Europe. As a consequence, the estimated values of the mechanical parameters (compressive and shear strength, elastic moduli) are generally high with decent compressive and seismic capacity of the investigated walls.

### 5.2. Type 2 (Cross Sectional Thickness ≥60 cm)

**Figure 17:**
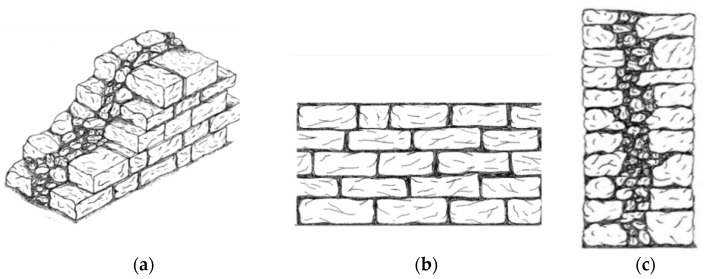
Sketches of a wall panel Type 2 (cross sectional thickness ≥60 cm): (**a**) axonometric view, (**b**) front view, (**c**) wall cross section.

The second type of masonry walls noted in Gjirokastër is characterized by a cross sectional thickness ≥60 cm (Figure 17). Again, two types of stone materials have been observed: the white limestone (letter designation J, Table 1) and the soft stone (letter designation L). Considering these two variables (wall thickness and stone material), in total, the main macro-typologies of walls of Gjirokastër used in this investigation are 4.

For stone wall having a cross sectional thickness ≥60 cm, the analysis of the cross section revealed a structure of three wall leaves: two outer parts typically made of cut stones, and an inner core made of peddles, small stones with a large percentage of voids. There are no transversal connections (headers) between the wall leaves and this highly reduces the wall’s capacity against seismic actions.

Typically, head joints are properly staggered (outcome PF), the mortar is lime-based, the horizontal bed joints are continuous (outcome F), the stones are worked, and their shape is parallelepiped (outcome F). It is worth noting that timber ties or beams sometimes used in the past to connect the wall leaves have not been considered in the assessment. Their presence could highly improve the structural response of a wall panel, and a case-by-case analysis is needed for those buildings where these timber elements are noted. For walls having a cross sectional thickness ≥60 cm, we have concluded that transversal connection (WC parameter) between wall leaves is weak (outcome: NF). 

Table 3 and Table 4 show the results of the visual analysis in terms of MQI indices and mechanical characteristics: for walls having a cross sectional thickness ≥60 cm, results are given for both J and L stone types. It can be noted that the use of a soft stone (Type L) highly affects the structural assessment, with a value of MQI index of 3.85 for horizontal in-plane and out-of-plane loading. Regardless of the thickness size, it is observed that the type of the stone has a higher influence on the structural behavior of the wall. The MQI values varies from 6.5–7.5 to 5.5–6.5 for stone J, whereas 4.55–5.25 to 3.85–4.55 for stone L.

Table 4 reports the values of the most important mechanical parameters resulting from the application of the MQI method for the four macro-typologies of walls noted in Gjirokastër. These parameters are given in terms of an average value: however, to take into account the intrinsic variability of a material like masonry, an “interval of confidence” is also given in parentheses in Table 4 for each mechanical parameter. The use of an interval is consistent with the method suggested by the Italian Seismic Code [39] and attached Guidelines [40]: in this code, the masonry mechanical parameters are given in terms of an interval of confidence, where the average value is typically the mean. 

A MQI value smaller than 4 is an indication of a likely masonry failure mode by disaggregation (The failure mode of a wall panel under the action of an earthquake can be categorized in two classes: masonry disaggregation and the development of a local or global mechanism of wall elements (macroelements). Under the action of an earthquake, some types of masonry are typically unable to deform and to split in macroelements and fail by “masonry disaggregation” or “masonry crumbling”. This failure is typical of some types of low-quality masonry: it is worth noting that the seismic capacity of a building where the governing failure mode is disaggregation is often low). This failure mode should always be avoided in masonry buildings and interventions are normally needed to reinforce the wall when this failure mode is likely to occur during a seismic event. 

For walls made of stone material type J, the outcomes of 6 out of the 7 parameters remained unchanged. However, the use of a stone of higher compressive and flexural strengths has a positive effect on the structural response of the walls: the MQI_V_, MQI_O_, MQI_I_ increased to 6.5, 5.5, and 5.5, respectively, and better results for the mechanical parameters have been estimated. 

**Figure 18:**
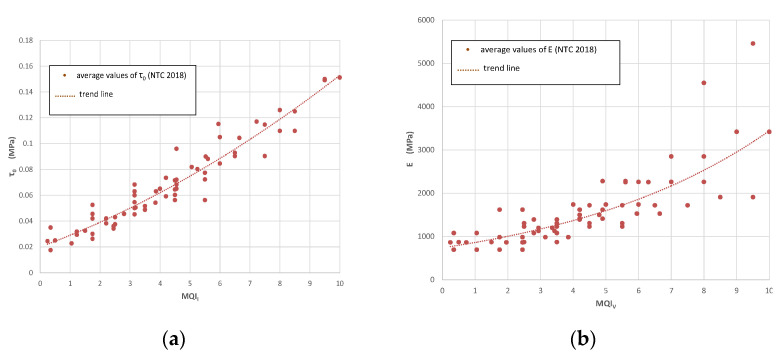

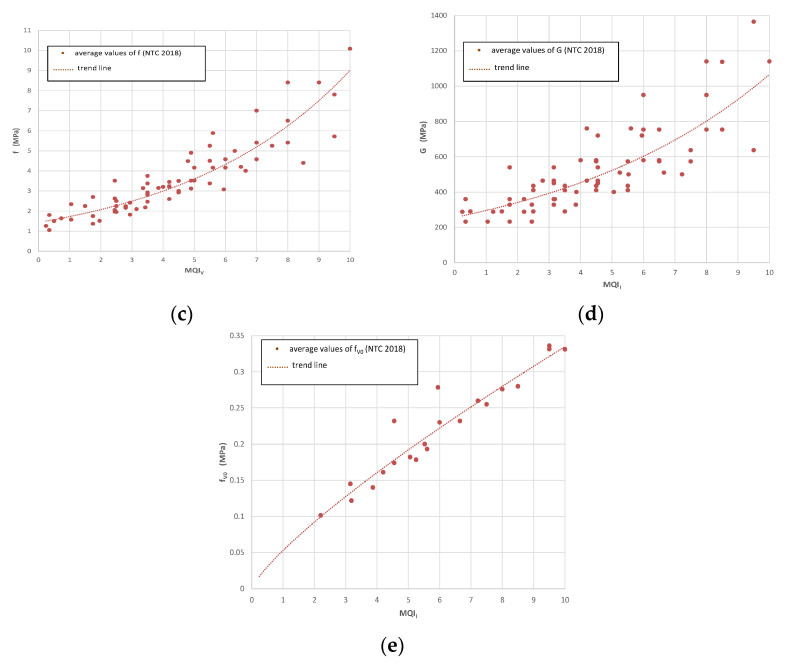
MQI correlations with masonry mechanical parameters: (**a**) Masonry shear strength τ_0_ vs. MQI_I_ (NTC 2018, Italian Seismic Code, [40]), (**b**) Masonry Young’s modulus E vs. MQI_V_, (**c**) Masonry compressive strength f vs. MQI_V_, (**d**) Masonry shear modulus G vs. MQI_I_, (**e**) Masonry shear strength f_V0_ vs. MQI_I_.

Figure 18 shows the correlation curves used to estimate the mechanical properties of the 4 wall typologies of Gjirokastër. These curves have been calibrated using the experimental data available in the scientific literature and suggested by the Italian Building Code (NTC 2018) [41].

## 6. Conclusions

In this paper the main characteristics of the traditional masonry constructions of the UNESCO heritage site of Gjirokastër, Albania, which is located in a high-risk seismic area on the seismogenic zone of Ionian Coast are discussed and presented. The construction materials used in Gjirokastër are roughly cut stones which although look similar in shape, color, and dimensions, exhibited significant difference in terms of physical and mechanical properties.

Identification of material types of these stone typologies resulted in specimen collection of samples in traditional stone quarries nearby. The stones were cut into regular prismatic shape as of relevant international standards and flexural, compressive strength, as well as porosity, water absorption, and apparent densities were computed.

From the test results it was observed that there is a significant scattering of the mechanical properties of the building stones. The compressive strength varies from 22.7 to 115 MPa, whereas flexural strength from 8 to 33 MPa. This fact is also supported by the different use of various stones in construction. There was also found a close relationship between the physical and mechanical properties.

The assessment of the structural behavior of the masonry buildings demonstrated that the shear walls used in Gjirokastër for construction were of acceptable quality: the stones, mainly stones with medium-to-very high mechanical properties, were typically roughly reduced and cut to prismatic shape. Unlike the stone masonry often used in Italy, Greece, and other neighboring countries, rubble or pebble stone masonry is very rare in Gjirokastër buildings.

The structural response of four stone masonry typologies was investigated using the Masonry Quality Index (MQI). The results showed that, in general, the failure of masonry is considered to be at an acceptable level. The MQI provided an estimation of the main mechanical parameters (compressive and shear strength, elastic moduli). However, only for one masonry typology (the thick masonry made of a soft stone), the MQI results were below the threshold and a risk of failure by disaggregation under the seismic action was observed.

For all other investigated masonry typologies, the MQI analysis provided an index result varying between 5.25–7.5 (MQI_V_), 4.9–7 (MQI_O_), and 4.55–6.5 (MQI_I_). Of particular interest are the results in terms of masonry shear strength, *τ*_0_, also in consideration of the high seismic hazard of the territory of Gjirokastër. The average masonry shear strength *τ*_0_, estimated using the correlation curves, resulted in 0.097 MPa (Type 1 masonry, J stone), 0.082 MPa (Type 1 masonry, L stone), and 0.069 MPa (Type 2 masonry, J stone). Based on the authors’ experience, these values are slightly higher compared to the average typical shear strength of historic masonry of other common masonry typologies studied and tested by the authors in the last two decades. 

## Figures and Tables

**Table 1:** The building stone coding according to their origin.

Acronym	Stone Type	Origin
D	Red stone	Dropulli Valley
J	White limestone	Jorgucat
G	Gushëpëllumb (Pigeon neck)	Dropulli Valley
L	Black stone	Lunxhëri & Poliçan
O	Grey stone	Odrie
P	Porous stone of Peshkepi	Peshkëpi
K	River stone	Kardhiq & Drino

**Table 2:** Numerical values of the seven parameters for the calculation of the MQI.

Parameters	Vertical Loading (V)			Horizontal Out-of-Plane Loading (O)			Horizontal In-Plane Loading (I)		
	NF	PF	F	NF	PF	F	NF	PF	F
HJ	0	1	2	0	1	2	0	0.5	1
WC	0	1	1	0	1.5	3	0	1	2
SS	0	1.5	3	0	1	2	0	1	2
VJ	0	0.5	1	0	0.5	1	0	1	2
SD	0	0.5	1	0	0.5	1	0	0.5	1
MM	0	0.5	2	0	0.5	1	0	1	2
SM	0.3	0.7	1	0.5	0.7	1	0.3	0.7	1

**Table 3:** The building stone coding according to their origin.

Wall Type	Stone	HJ	WC	SS	VJ	SD	MM	SM	MQI_V_	MQI_O_	MQI_I_
Type 1	Stone J	F	PF	F	PF	PF	PF	F	7.5	7	6.5
	Stone L	F	PF	F	PF	PF	PF	PF	5.25	4.9	4.55
Type 2	Stone J	F	NF	F	PF	PF	PF	F	6.5	5.5	5.5
	Stone L	F	NF	F	PF	PF	PF	PF	4.55	3.85	3.85

**Table 4:** Results of the assessment of the mechanical properties of the masonry materials.

Wall Type	Stone	Compressive Strength, *f*, (MPa)	Shear Strength, *τ*_0_ (MPa) [44]	Shear Strength, *f*_0_, (MPa) [44]	Shear Modulus, *G*, (MPa)	Young’s Modulus, *E*, (MPa)
Type 1	Stone J	5.69 (4.53–6.68)	0.097 (0.070–0.121)	0.237 (0.167–0.306)	647 (538–756)	2316 (538–756)
	Stone L	4.74 (3.72–5.74)	0.082 (0.060–0.102)	0.207 (0.145–0.269)	561 (466–656)	2011 (1668–2353)
Type 2	Stone J	3.77 (2.92–4.60)	0.069 (0.052–0.069)	0.178 (0.124–0.232)	490 (407–573)	1659 (1374–1944)
	Stone L	3.31 (2.55–4.07)	0.060 (0.045–0.074)	0.156 (0.108–0.203)	443 (368–519)	1490 (1232–1746)

## Date Availability Statement

Data is contained within the article.
